# Spatial distribution characteristics and analysis of influencing factors on different manufacturing types in Shandong Province

**DOI:** 10.1371/journal.pone.0291691

**Published:** 2023-09-20

**Authors:** Yanghua Zhang, Qiwen Zheng, Shuai Ye, Kewei Zhang, Weipeng Lin

**Affiliations:** School of Architecture and Urban Planning, Shandong Jianzhu University, Jinan, China; National University of Sciences and Technology, PAKISTAN

## Abstract

Investigating the spatial distribution characteristics and influencing factors of various industry types is critical for promoting the high-quality transformation and development of China’s industry. This study combined the Getis-Ord Gi* statistic method, the random forest-based importance assessment method, and the geographically weighted regression method to determine the spatial distribution characteristics of four industry types and their influencing factors. The results revealed that the raw material industry was primarily concentrated in the surrounding districts and counties of Linyi and Qingdao. The food and light textile industry was mainly concentrated in the surrounding districts and counties of Qingdao, and a few were concentrated in some counties of Linyi. The processing and manufacturing industry was also concentrated in the surrounding districts and counties of Qingdao, and a few were concentrated in the belt regions connecting Jinan, Zibo, and Weifang. The high-tech industry was mainly concentrated in the surrounding districts and counties of Jinan and Qingdao. The key spatial influencing factors of the four industry types were different. The number of employees in the secondary industry and road density were most important in determining the spatial distribution of the raw material industry. The financial environment and number of research institutions were most important to the spatial distribution of the food and light textile industry. The gross domestic product and number of medical facilities were most important to the spatial distribution of the processing and manufacturing industry. Urbanization rate, number of research institutions, and gross domestic product were most important to the spatial distribution of the high-tech industry. Geographically weighted regression analysis revealed that the impact intensity of these key factors on the industry exhibits significant spatial heterogeneity. Taken together, these results are useful for formulating the development strategy for each industrial type in different regions.

## Introduction

China’s industry has been developing rapidly since the reform and opening up, and most cities are completely industrialized [1]. However, the development process is characterized by high resource consumption and substantial environmental pollution [2, 3]. To improve the development process, the government has implemented a number of policies to guide industrial transformation and upgrades [4, 5]. Therefore, research into the spatial distribution characteristics and influencing factors of various industrial types is critical for promoting the high-quality transformation and development of China’s industries.

Many studies on industrial spatial distribution characteristics have been conducted in recent years. These studies can be classified into macro, meso, and micro scales based on the size of the research unit [6, 7]. A macroscale study analyzes the industrial spatial characteristics of the whole country or urban agglomeration using a province or city as the analysis unit [8]. A mesoscale study analysis unit is typically a county or industrial park [4]. The enterprise plot or enterprise point is commonly used as the analysis unit in microscale studies and are used to investigate the spatial agglomeration characteristics of different types of industry enterprises [9, 10]. Macroscale and mesoscale studies primarily use the administrative region as the analysis unit, and the research data primarily consist of socioeconomic statistics and regional survey data, making these types of studies more feasible due to the ease with which data can be collected. On the other hand, microscale studies have fewer administrative restrictions, and enterprise spatial distribution characteristics and positions can be analyzed at a fine scale. However, obtaining fine-scale data is difficult. Previous microscale studies relied primarily on historical planning maps or enterprise network data containing land use area, position, and management conditions [6, 11]. With the development of big data in recent years, point of interest (POI) data have been widely used in microscale enterprise studies, and it is expected that this trend will continue in the future [2, 12, 13].

In terms of the research industry type and structure, some studies focus on one industry type while others focus on multiple industry types; some focus on secondary industries, and some focus on tertiary industries [14–16]. For example, Shiping used the electric power industry in the Yellow River Basin as a research object and studied its spatiotemporal development pattern and green development path [17]. Li took 31 different manufacturing types as research objects and used the industrial location quotient to investigate the spatial agglomeration characteristics of different manufacturing types [18]. Zhang used the solar photovoltaic industry as a research object and analyzed its spatial agglomeration characteristics in China [19]. Fu analyzed the market and industrial agglomeration impacts on the development of the furniture industry using the furniture industry as a research object [15].

Many factors influence industrial spatial distribution characteristics, including economic, policy, traffic, and location factors [20]. Zhang concluded that government intervention, density of road networks, and land marketization are critical factors that should be given greater consideration in government industrial policymaking [8]. Yan discovered that traffic accessibility, land price, and spatial agglomeration have a significant effect on the spatial distribution characteristics of the accommodation industry in Beijing [21]. Che found that the region’s gross domestic product (GDP), population size, per capita disposable income of residents, comprehensive accessibility of cities, and proportion of tertiary industry have a significant impact on the spatial distribution of wholesale and retail outlets and restaurants [22]. Wang analyzed the spatial-temporal patterns and influencing factors of the hotel industry in Hunan province over a period of 40 years and found that its spatial distribution pattern showed a transformation trend from a traffic and business district-dependent type to a tourist resource and college-dependent type [23].

In summary, many valuable studies on the spatial distribution and influencing factors on industry have been conducted. However, it is unclear whether there are any differences in their distribution characteristics between industrial types, particularly high-end manufacturing and low-end manufacturing, what the possible influencing factors might be, and the degree of influence of each factor. To date, only a few studies have focused on these issues. This study focuses on the disclosure of spatial distribution characteristics and the analysis of its influencing factors on different manufacturing types using a large amount of POI data and the Getis-Ord Gi* statistical analysis and geographically weighted regression (GWR) methods.

The remainder of this paper is organized as follows. The next section describes the study area and data. The third section describes the spatial distribution disclosure and influencing factor analysis algorithms for different industries. The fourth section reports the results of each research procedure, and the fifth section discusses the research results. The sixth section concludes the paper.

## Study area and data

**Fig 1:**
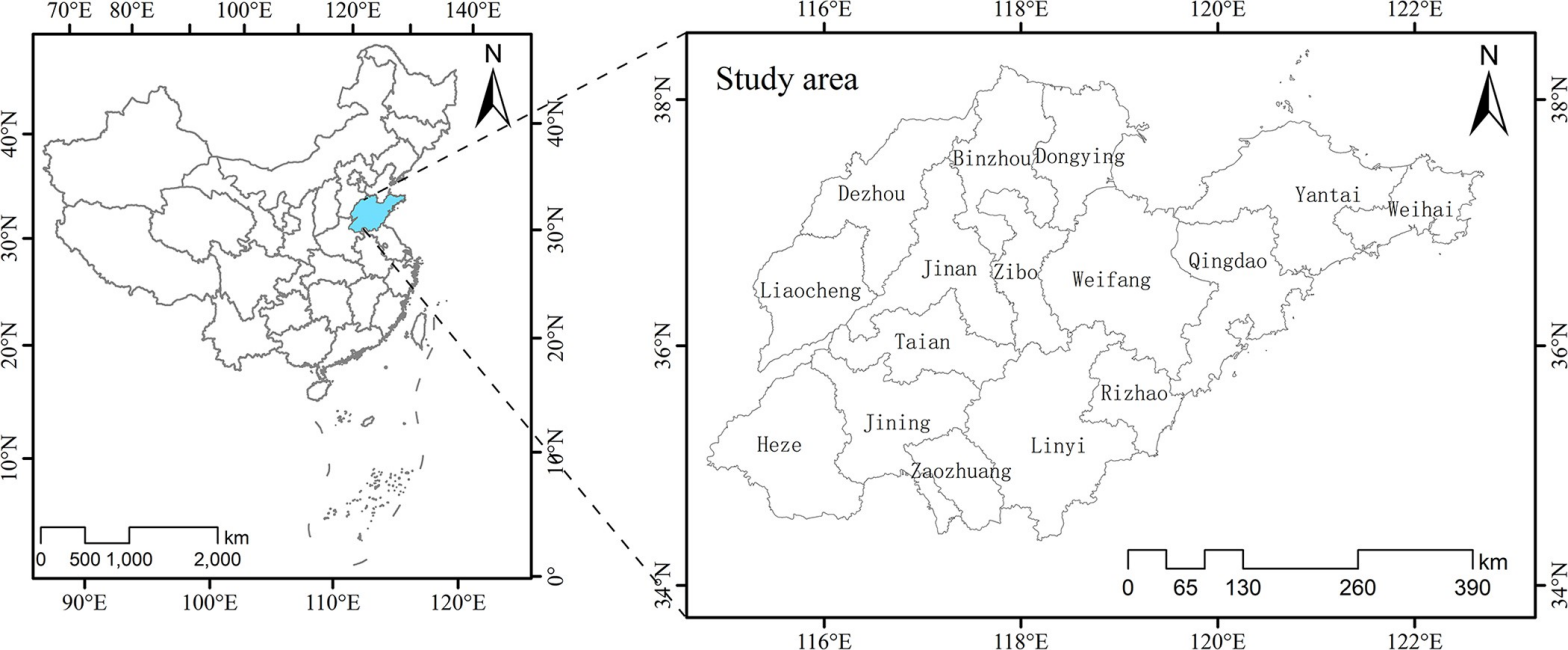
The study area.

Shandong Province was selected as the study location (**Fig 1**). After reforming and opening, the economy of the Shandong Province grew rapidly, economic structure reform deepened, economic and social development underwent major changes, and the industrial structure greatly improved [24]. In recent years, the economy of Shandong has consistently ranked third in China, with the chemical industry economy ranking first [25] and the textile industry ranking third [26]. However, owing to historical factors, national macroeconomic policies, environmental effects, and international economic environmental constraints, the Shandong Province’s industrial structure remains problematic [24]. For example, the proportion of technology-intensive industrial manufacturing is low, while the proportion of the intermediate inputs industry of iron and steel industry and non-metallic materials are high. The proportion of manufactured consumer goods is close to the global average, but is higher in the textile industry and lower in the food industry. The raw materials industry has a higher proportion of resource consumption and pollution emissions than the world average. Furthermore, the spatial distribution of various industrial structures is uneven [24]. Given these circumstances, industrial transformation, upgrading, and high-quality development in Shandong Province are critical. Therefore, research on the spatial agglomeration characteristics of various industry types in Shandong Province is required.

In order to analyze the spatial distribution of different manufacturing types, approximately 500,000 points of interest (POI) from manufacturing, financial institution, research institution, medical facilities, railway station, locations in 2020 were collected using the application programming interfaces from the Baidu online map service provider. After filtering out points belonging to a unified institution but with different names, the accuracy of remaining points can be achieved 91% through 500 random sample tests. The administrative boundary data were obtained from the Resource and Environment Science and Data Center of the Chinese Academy of Sciences (https://www.resdc.cn/). The data of some influence factors were also collected from multiple sources in order to reveal the influence factors of manufacturing distribution. Specifically, 2019 county-level statistical yearbook data, the latest open street map (OSM) road network data (https://www.openstreetmap.org/), and digital elevation model (DEM) data from the Geospatial Data Cloud website (http://www.gscloud.cn/) were included. All spatial data were clipped to the same extent as that of the study area and the projection coordinate system was uniformly set to WGS_1984_UTM_Zone_50N.

## Methodology

### Manufacturing classification

Referring to China’s 2017 National Economic Industry Classification Standard, 2017 High Tech Industry Classification Standard, and some existing studies [27–29], this study chose four industry types for analysis: the food and light textile industry (FLT), raw material industry (RM), processing and manufacturing industry (PM), and high-tech industry (HT). The FLT mainly comprises several types of industries producing a diverse range of food products, textile products, and residential consumer products, such as the brewing, shoemaking, and furniture industries [28, 30]. The RM mainly includes industries that process raw materials of industrial and daily products, such as the wood-processing industry, metal-processing industry, petroleum refineries, and steel industry [31]. The PM mainly includes the manufacturing of mechanical products, communication equipment, and transportation products, including the automotive and shipbuilding industries. The HT refers primarily to China’s 2017 HT Classification Standard and includes the pharmaceutical industry, aerospace manufacturing industry, electronic information equipment manufacturing industry, and others [32]. The specific manufacturing types for each of the four industry types are shown in **Table 1**.

**Table 1:** Classification of different industry types.

Industry type	Detailed industry and manufacturing types
Food and light textile industry (FLT)	Agricultural and sideline food processing industry; Food manufacturing industry; Liquor, beverage, and refined tea manufacturing industry; Tobacco industries; Textile industry; Textile and clothing industry; Leather, fur, feathers and their products, and footwear industry; Furniture manufacturing industry; Paper and paper products industry; Printing and recording media reproduction industry; Cultural and educational, industrial and artistic, sports and entertainment equipment manufacturing industry.
Raw material industry (RM)	Wood processing and wood, bamboo, rattan, palm, and grass products; Petroleum processing, coking, and nuclear fuel processing industries; Chemical raw material and chemical product manufacturing industry; Chemical fiber manufacturing industry; Rubber and plastic products industry; Nonmetallic mineral products industry; Ferrous metal smelting and rolling processing industry; Nonferrous metal smelting and rolling processing industry; Fabricated metal products.
Processing and manufacturing industry (PM)	General equipment manufacturing industry; Special equipment manufacturing industry; Automotive industry; Railway, shipbuilding, aerospace, and other transportation equipment manufacturing industries; Electrical machinery and equipment manufacturing industry.
High-tech industry (HT)	Information chemicals manufacturing; Pharmaceutical industry; Manufacturing of copying and offset printing equipment; Manufacturing of calculators and currency specific equipment; Manufacturing of specialized equipment for the electronic industry; Medical equipment and device manufacturing; Aviation, spacecraft, and equipment manufacturing industry; Manufacturing of optical fibers and cables; Lithium ion battery manufacturing; Computer, communication, and other electronic equipment manufacturing industry; Instrument manufacturing industry; Aerospace repair.

The manufacturing types for each industrial POI was grouped by comparing its enterprise name with the name of the detailed industry and manufacturing types in **Table 1**. First, some POIs were automatically grouped by matching strings of the enterprise name and industry type name. Then, for POIs that could not be automatically classified, manual screening methods were used to individually determine the industry type. Finally, the classification quality of the POIs for each industry type was validated by manually checking 500 randomly sampled POIs. The accuracy levels of RM, FLT, PM, and HT were 91.2%, 93.1%, 90.3%, and 90.7%, respectively.

### Spatial distribution characteristic analysis

In spatial agglomeration analysis, the Getis-Ord Gi* statistic method, also called the hotspot analysis method, is widely used [33, 34]. The results show where features with high or low values cluster spatially. It works by examining each feature in the context of its neighbors. If the local sum of a feature and its neighbors is significantly larger than the sum of all other features, the feature has a high value and is surrounded by other features with high values. Thus, the feature and its neighbors will most likely be statistically classified as a hot region. The calculation formula is as follows:

$$G_{i}^{*}=\frac{\sum_{j=1}^{n}w_{i,j}x_{j}-\bar{X}\sum_{j=1}^{n}w_{i,j}}{S\sqrt{\frac{\left[n\sum_{j=1}^{n}w_{i,j}^{2}-{\left(\sum_{j=1}^{n}w_{i,j}\right)}^{2}\right]}{n-1}}},$$ (1)

where *x*_*j*_ is the attribute value for feature *j*, *w*_*i*,*j*_ is the spatial weight between features *i* and *j*, *n* is the total number of features,

$$\bar{X}=\frac{\sum_{j=1}^{n}x_{j}}{n},$$ (2)

and

$$S=\sqrt{\frac{\sum_{j=1}^{n}x_{j}^{2}}{n}-{\left(\bar{X}\right)}^{2}}.$$ (3)

### Key influencing factor analysis

A random forest (RF) model-based factor importance evaluation method was used to investigate the key factors that influence the spatial distribution of the four industry types. RF is an ensemble algorithm that generates many classification- and regression-like trees; each tree is grown with a different bootstrapped sample of the training data, and approximately one-third of the training data is omitted in the construction of each tree [35]. The performance of the RF is typically evaluated by the “out-of-bag” error (OOB error) analysis using withheld training data [36, 37]. The basic concept of RF-based factor importance is to calculate the increase in the OOB error after permuting the factor variable. The importance index is defined as follows:

$${IncMSE}_{x}=\frac{\sum_{i=1}^{N}\left|{error}_{2}-{error}_{1}\right|}{N},$$ (4)

where *IncMSE*_*x*_ is the importance evaluation index of factor *x*, *N* is the number of RF trees, *error*_2_ is the OOB error after permuting factor values, and *error*_1_ is the OOB error before permuting factor values. A higher *IncMSE*_*x*_ value indicates a greater importance of factor x. In addition, two important parameters have to be selected for the RF analysis: the number of trees (Ntree) and the number of randomly selected factors at each node (Mtry) [37]. At present, most studies have generally set Ntree to the default number of 500 and Mtry to the square root of the number of input factors and they have achieved ideal performance [36]. Therefore, this study also refers to these existing studies on the determination of these parameters. The selected factors are shown in **Table 2**.

**Table 2:** Description of the influence factors.

factors	Description	Sources	Influence relationship
UR	Urbanization rate	Statistical yearbook of each county (2019)	+
GDP	Gross domestic product of each county or district	Statistical yearbook of each county (2019)	**+**
FE	Number of financial institutions	Extracted from POI of financial institutions in 2020	+
NES	Number of employees in secondary industry	Statistical yearbook of each county (2019)	**+**
NRI	Number of research institutions	Extracted from POI of research institutions in 2020	**+**
NMF	Number of medical facilities	Extracted from POI of the medical facilities in 2020	**+**
RD	Road density	Latest open street map (OSM) road network data	**+**
DRS	Distance from railway station	Extracted from POI of the railway stations in 2020	**-**
VP	Vegetation proportion	Statistics from the 10m land cover data of [40]	-
WP	Water proportion	Statistics from the 10m land cover data of [40]	-
Slope	The degree of steepness of the terrain	Extracted from digital elevation model (DEM) data	-
NDZ	Number of development zones	Statistical yearbook of each city (2019) and network resources	+

#### Urbanization rate (UR)

In China, urbanization is a major driver of economic development [8]. It also indirectly promotes industrial development and upgrading. In this study, we extracted the proportion of the urban population as the urbanization rate from the statistical yearbook of each county.

#### Gross domestic product (GDP)

GDP measures a city’s total economic output. High-GDP regions are more likely to attract more talent and capital agglomeration [38], which in turn promotes industry upgrading.

#### Financial environment (FE)

Industrial development cannot be separated from financial capital support [39]. The type and number of financial institutions in a region can reflect its financial environment to some extent. Convenient and multi-source financial assistance helps to promote industrial development.

#### Labor

For the labor factor, the number of employees in the secondary industry (NES) was selected. According to the theory of industrial location, labor is an important driving force for industrial location selection [20].

#### Research environment (RE)

The timely transformation of scientific research achievements is critical for industry development and upgrading. The primary sectors that produce scientific research achievements are research institutions. In this study, the number of research institutions (NRI) in one region was chosen as its research environment.

#### Medical conditions (MC)

The condition of public service facilities influences talent and capital agglomeration. A medical facility is an essential public service facility. This study selected the number of medical facilities (NMF) as the medical condition factor.

#### Traffic factors

According to the theory of industrial location, traffic conditions are an important driving force for industrial location selection [20]. This study selected road density (RD) and the distance from a railway station (DRS) as traffic factors.

#### Ecological factors

Sustainable development is currently an important theme worldwide. For many years, China has been committed to environmental protection as part of its economic development. To some extent, most forms of industry development conflict with ecological protection. The vegetation proportion (VP) and water proportion (WP) of one region were chosen as ecological factors in this study.

#### Topographical factor

Topographic conditions have a significant impact on the selection of industrial location. Therefore, slope was selected as the topographic factor in this study.

#### Economic development zones

Economic development zones are a government initiative to promote industrial development. It can achieve infrastructure sharing and agglomeration development among different industries. As a result, regions with development zones benefit from industrial development. In this study, the number of development zones (NDZ) in each region was selected.

### Geographically weighted regression (GWR) analysis

The first law of geography states that adjacent geographical entities are more similar [41]. Meanwhile, interregional spatial correlation and spatial heterogeneity exist as a result of the unequal distribution of natural resources and socioeconomic factors in different regions. Therefore, using a global regression model to determine the relationship between multiple spatial variables is impossible. To account for spatial heterogeneity when analyzing the impact of multiple factors on industrial spatial distribution, the geographically weighted regression (GWR) method was used to reveal the spatially varying relationships between manufacturing types (dependent variable) and key influencing factors (independent variables) [42]. The GWR model can be expressed as

$$y(u_{0})=\beta_{0}(u_{0})+\sum_{j=0}^{k}\beta_{j}(u_{0})x_{j}(u_{0})+\varepsilon (u_{0}),$$ (5)

where *x*_*j*_(*u*_0_) and *ε*(*u*_0_) are the value of the *j*-th independent variable and the residual at location *u*_0_, respectively. *β*_0_(*u*_0_) and *β*_*j*_(*u*_0_) are the intercept and estimated regression coefficient for the j-th independent variable at location *u*_0_, respectively. Parameters *β*_0_(*u*_0_) and *β*_*j*_(*u*_0_), which vary over spatial locations, can be estimated by solving a weighted matrix equation:

$$\beta (u_{0})={\left(X^{T}W(u_{0})X\right)}^{-1}X^{T}W(u_{0})Y,$$ (6)

where *W* denotes the spatial weight matrix, the selection and setting of which are a key consideration in GWR. It is calculated in two major steps. The first step is the selection of a proper kernel function to express the spatial relationship between the units. Specifically, four major kernel functions are used in the existing research: fixed Gaussian, fixed bi-square, adaptive bi-square, and adaptive Gaussian. Because the merits of a kernel function play a direct and decisive role in obtaining the most accurate possible regression parameter estimation of spatial heterogeneity, fixed Gaussian was chosen as the kernel function in this study after careful analysis and comparison. It is expressed as:

$$w_{ij}=\exp\left(-\frac{d_{ij}^{2}}{\theta^{2}}\right),$$ (7)

where *w*_*ij*_ represents the distance weight from sample *i* to sample *j*; *d*_*ij*_ is the Euclidean distance between samples *i* and *j*; and *θ* is the bandwidth, which determines the rate at which the spatial weight attenuates with distance.

The second step of the spatial weight matrix calculation is the selection of the optimal bandwidth, which can contribute to a higher fitting degree. The Akaike Information Criterion corrected (AICc) method was chosen for optimal bandwidth selection in this study.

## Results

### Spatial distribution characteristics of different industry types

**Fig 2:**
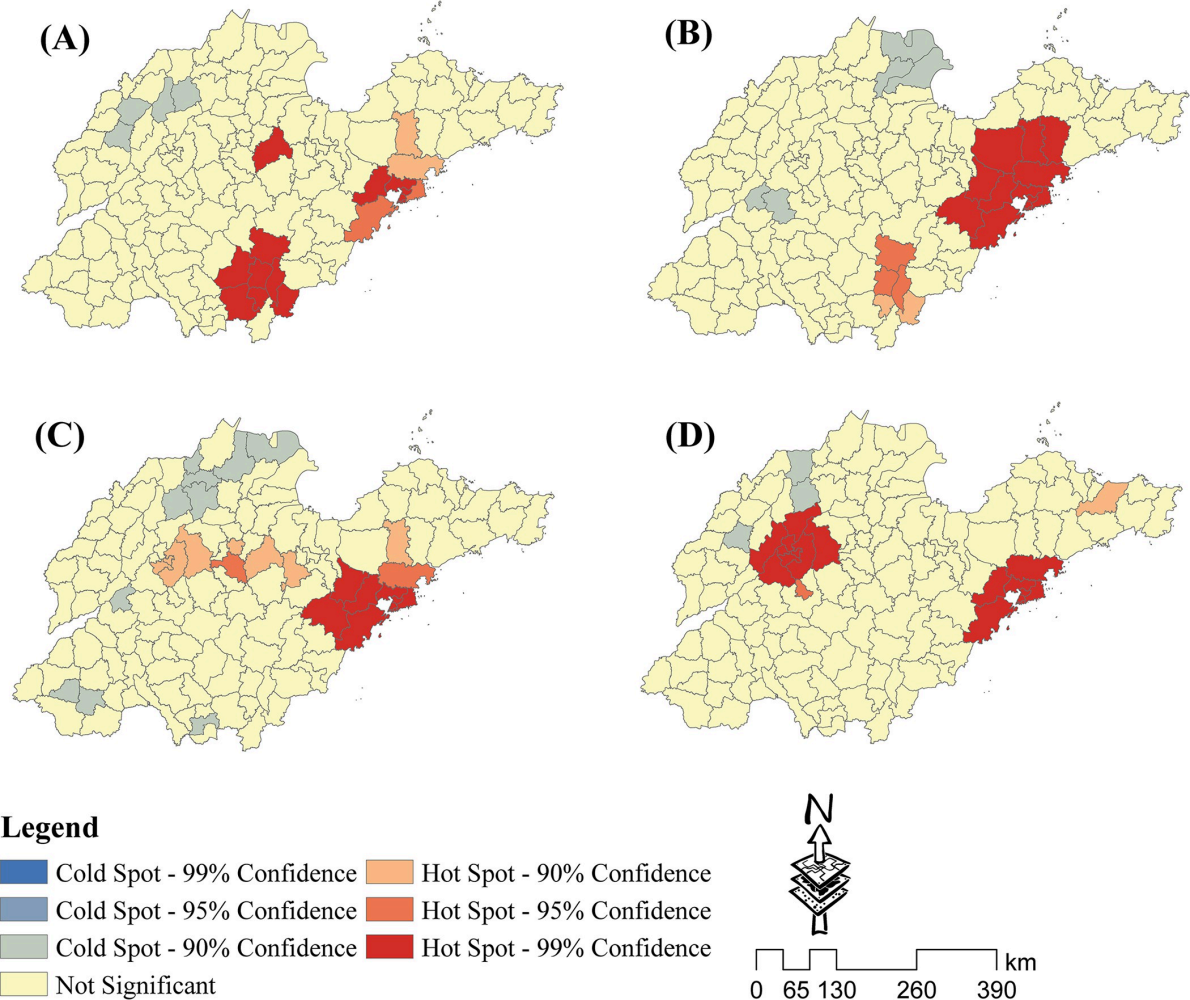
The Getis-Ord Gi* statistical analysis of the four industry types. Analysis results for (A) the raw material industry (RM), (B) food and light textile industry (FLT), (C) processing and manufacturing industry (PM), and (d) high-tech industry (HT).

The spatial distribution characteristics for each industry are shown in **Fig 2**. The four industry types have different agglomeration distribution characteristics, and are primarily concentrated in a few regions. Most regions lack significant industrial agglomeration distribution characteristics. The RM hotspot regions were mainly concentrated in the surrounding districts and counties of Linyi and Qingdao (**Fig 2**A), and the coldspot regions were mainly concentrated in four counties of Dezhou city in northern Shandong. The FLT hotspot regions were primarily concentrated in the surrounding districts and counties of Qingdao (**Fig 2**B), and a few were concentrated in some counties of Linyi. The FLT coldspot regions were mainly concentrated in the Yellow River estuary area in Dongying City. The PM hotspot regions were primarily concentrated in the surrounding districts and counties of Qingdao (**Fig 2**C), and a few were concentrated in the belt regions connecting Jinan, Zibo, and Weifang. The PM coldspot regions were mainly concentrated in a few counties in northern Shandong Province. The HT hotspot regions were primarily concentrated in the surrounding districts and counties of Jinan and Qingdao (**Fig 2**D). The HT coldspot regions were mainly concentrated in three counties in northern Shandong Province.

### Key influence factor analysis results

**Fig 3:**
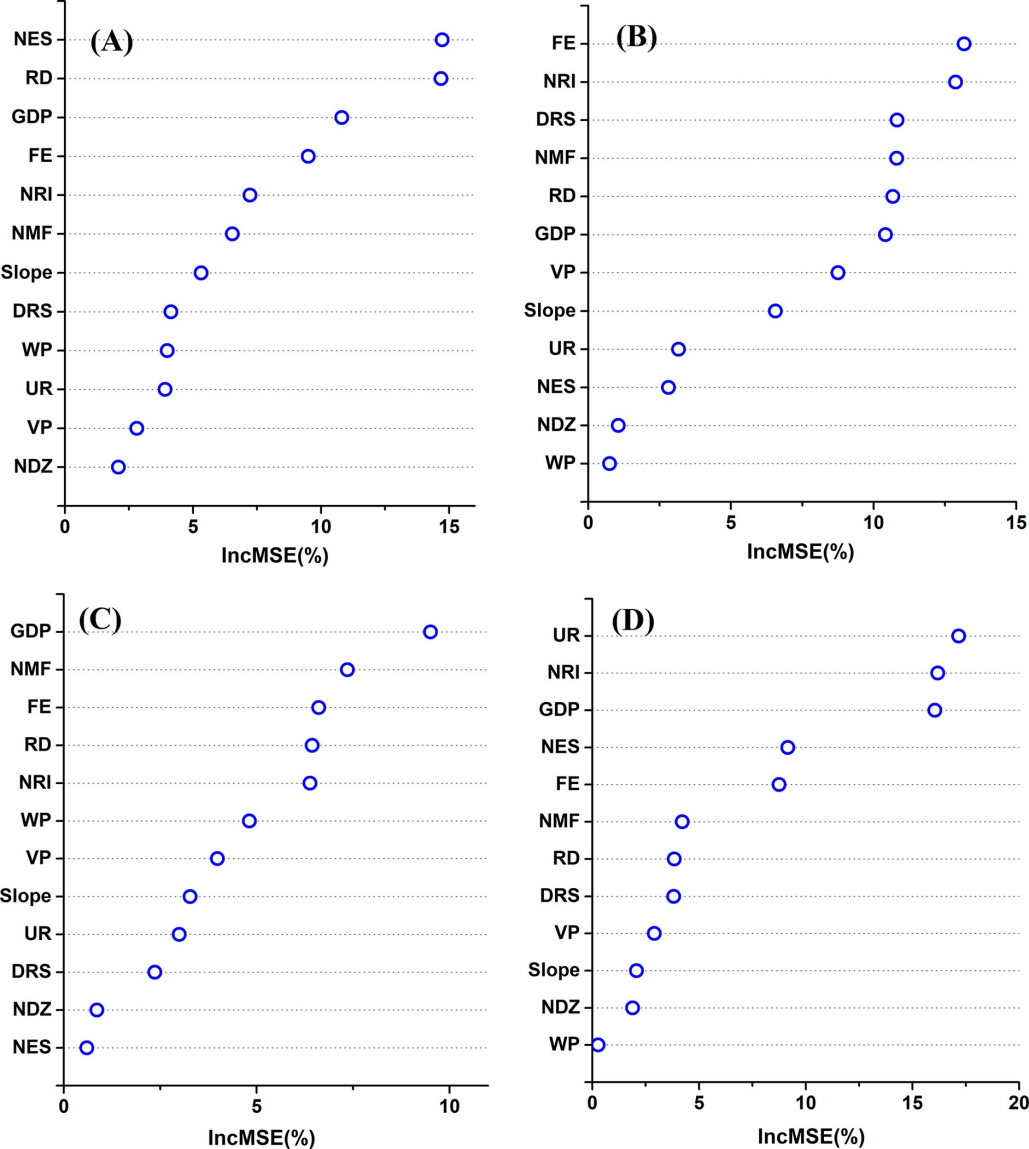
The importance of factors influencing the spatial distribution characteristics of different industry types. Evaluation results of (A) the raw material industry (RM), (B) food and light textile industry (FLT), (C) processing and manufacturing industry (PM), and (D) high tech industry (HT). UR: urbanization rate; GDP: gross domestic product; FE: number of financial institutions; NES: number of employees in secondary industry; NRI: number of research institutions; NMF: number of medical facilities; RD: road density; DRS: distance from railway station; VP: vegetation proportion; WP: water proportion; Slope: the degree of steepness of the terrain; NDZ: number of development zones.

The importance of factors influencing the spatial distribution characteristics of different industry types are shown in **Fig 3**. The key influencing factors for various industries differ. NES played the most important role in the spatial distribution of the RM, with RD, GDP, and FE ranked second, third, and fourth, respectively (**Fig 3**A). This indicates that labor and transportation factors are most important for the RM development.

FE played the most important role in the spatial distribution of the FLT, with NRI, DRS, and NMF ranked second, third, and fourth, respectively (**Fig 3**B), indicating that production service facilities and research and development facilities are most important for FLT development. GDP played the most important role in the spatial distribution of the PM, with NMF and FE ranked second and third, respectively (**Fig 3**C). These results indicate that economic and public service facilities are most important for PM development.

UR played the most important role in the spatial distribution of the HT, with NRI and GDP ranked second and third, respectively (**Fig 3**D). These results suggest that the urban development level and research and development facilities are most important for HT development.

### GWR analysis results

A GWR analysis was performed to investigate the relationship between the key factors and industry type. The top four ranked influencing factors from each industry type were selected for GWR analysis, and the regression effect is shown in **Table 3**. The values of R^2^ and adjusted R^2^ of the four manufacturing types are all > 0.5, and the values of the residual squares are all < 2.1. These results indicate that the regression analog effect is positive.

**Table 3:** GWR analysis of the four industry types.

	RM	FLT	PM	HT
R^2^	0.724	0.845	0.598	0.788
Adjusted R^2^	0.647	0.761	0.539	0.744
AICc	-301.84	-278.63	-151.05	-243.74
Residual Squares	0.561	0.470	2.052	0.95

RM: raw material industry; FLT: food and light textile industry; PM: processing and manufacturing industry; HT: high-tech industry; AICc: Akaike Information Criterion corrected.

**Fig 4:**
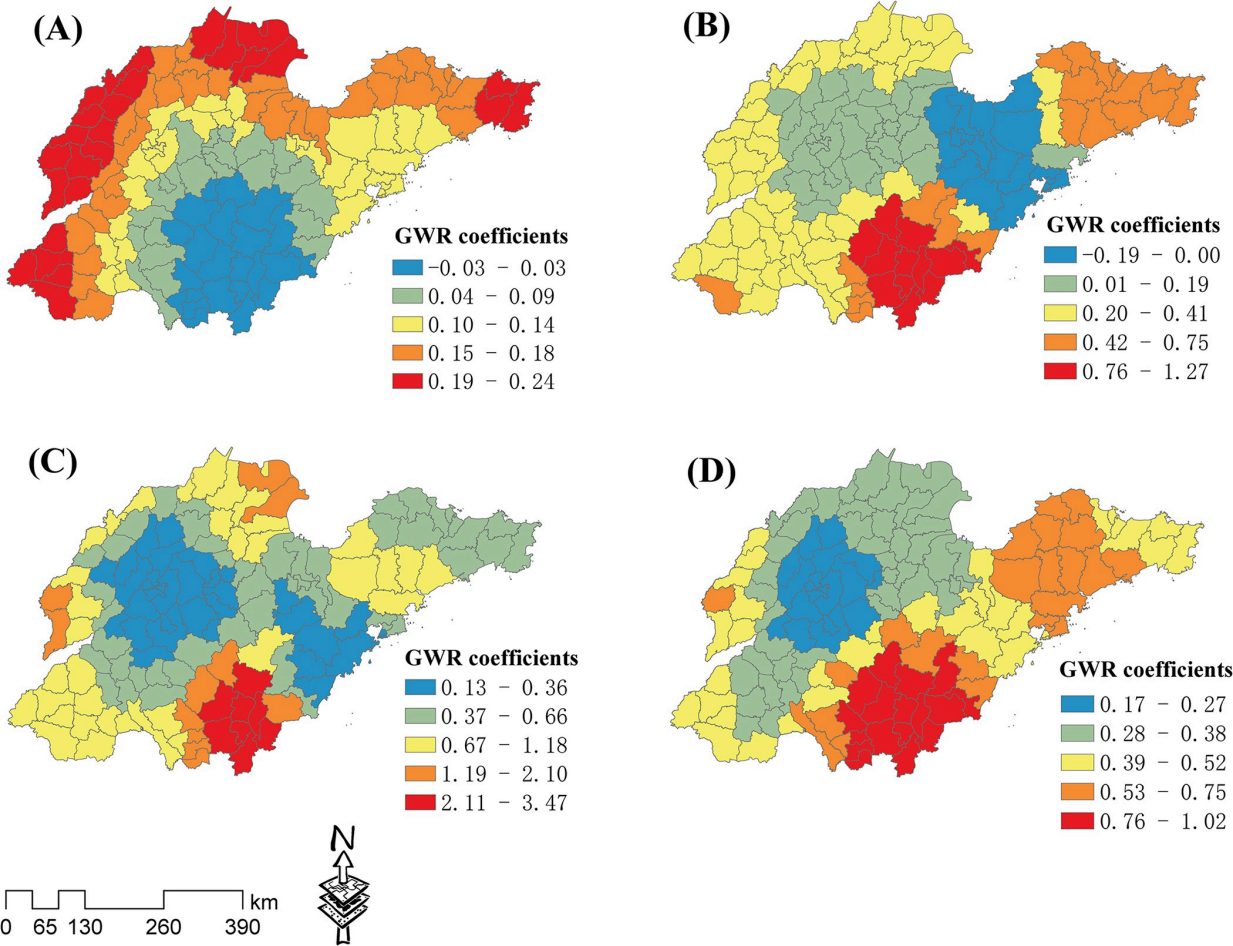
Local GWR coefficients of the raw material industry (RM) and key influence factors. Analysis results for (A) number of employees in secondary industry (NES), (B) road density (RD), (C) gross domestic product (GDP), and (D) number of financial institutions (FE).

The local GWR coefficients of the RM and the key influence factors of NES, RD, GDP, and FE are shown in **Fig 4**. The regression coefficient value ranges of NES, RD, GDP, and FE were -0.03–0.24, -0.19–1.27, 0.13–3.47, and 0.17–1.02, respectively, indicating that GDP had the strongest impact on the spatial distribution of the RM. The NES influence intensity gradually increased from south to northwest and northeast (**Fig 4**A). RD had a negative impact on the RM in the western Shandong Peninsula, and its influence intensity increased from the middle regions to the southern and eastern peninsula regions (**Fig 4**B). The GDP influence intensity gradually increased from the middle regions to the southern regions and a few northeastern regions (**Fig 4**C). The FE influence intensity gradually increased from the northwest to the southern and eastern regions (**Fig 4**D).

**Fig 5:**
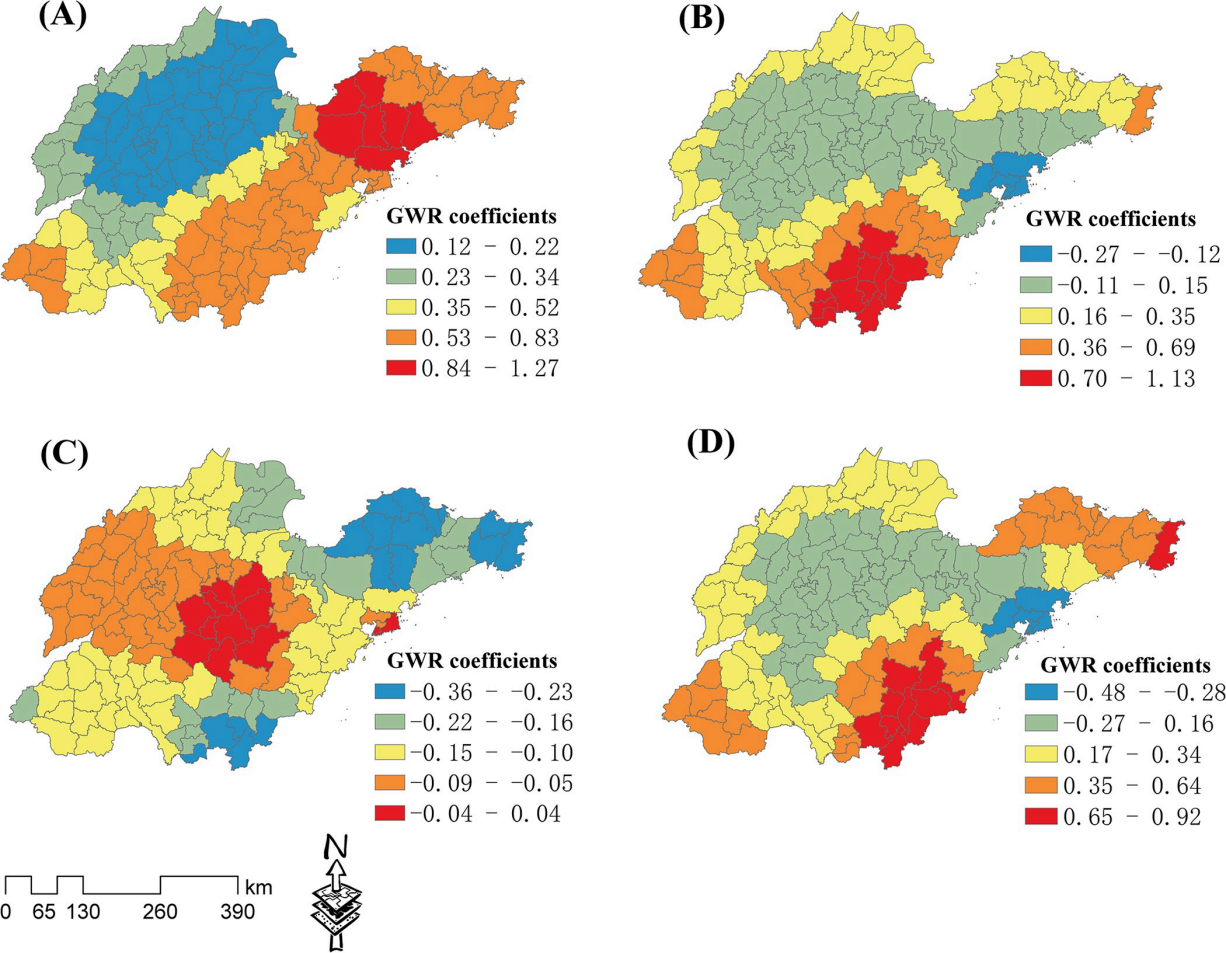
Local GWR coefficients for the food and light textile industry (FLT) and key influence factors. Analysis results for (A) number of financial institutions (FE), (B) number of research institutions (NRI), (C) distance from railway station (DRS), and (D) number of medical facilities (NMF).

The local GWR coefficients of the FLT and the key influence factors of FE, NRI, DRS, and NMF are shown in **Fig 5**. The regression coefficient value ranges of FE, NRI, DRS, and NMF were 0.12–1.27, -0.27–1.13, -0.36–0.04, and -0.48–0.92, respectively, indicating that the FE had the strongest impact on the spatial distribution of the FLT. The FE influence intensity gradually increased from the north to the southeast and eastern peninsula regions (**Fig 5**A). The NRI influence intensity increased from the central and northern regions to the southern regions (**Fig 5**B). The DRS primarily had a negative impact on the FLT, and its influence intensity gradually increased from the central regions to the Shandong Peninsula and the southern regions (**Fig 5**C). The NMF had a significant negative impact on the FLT around Qingdao, and its positive influence intensity gradually increased from the central region to the south and east (**Fig 5**D).

**Fig 6:**
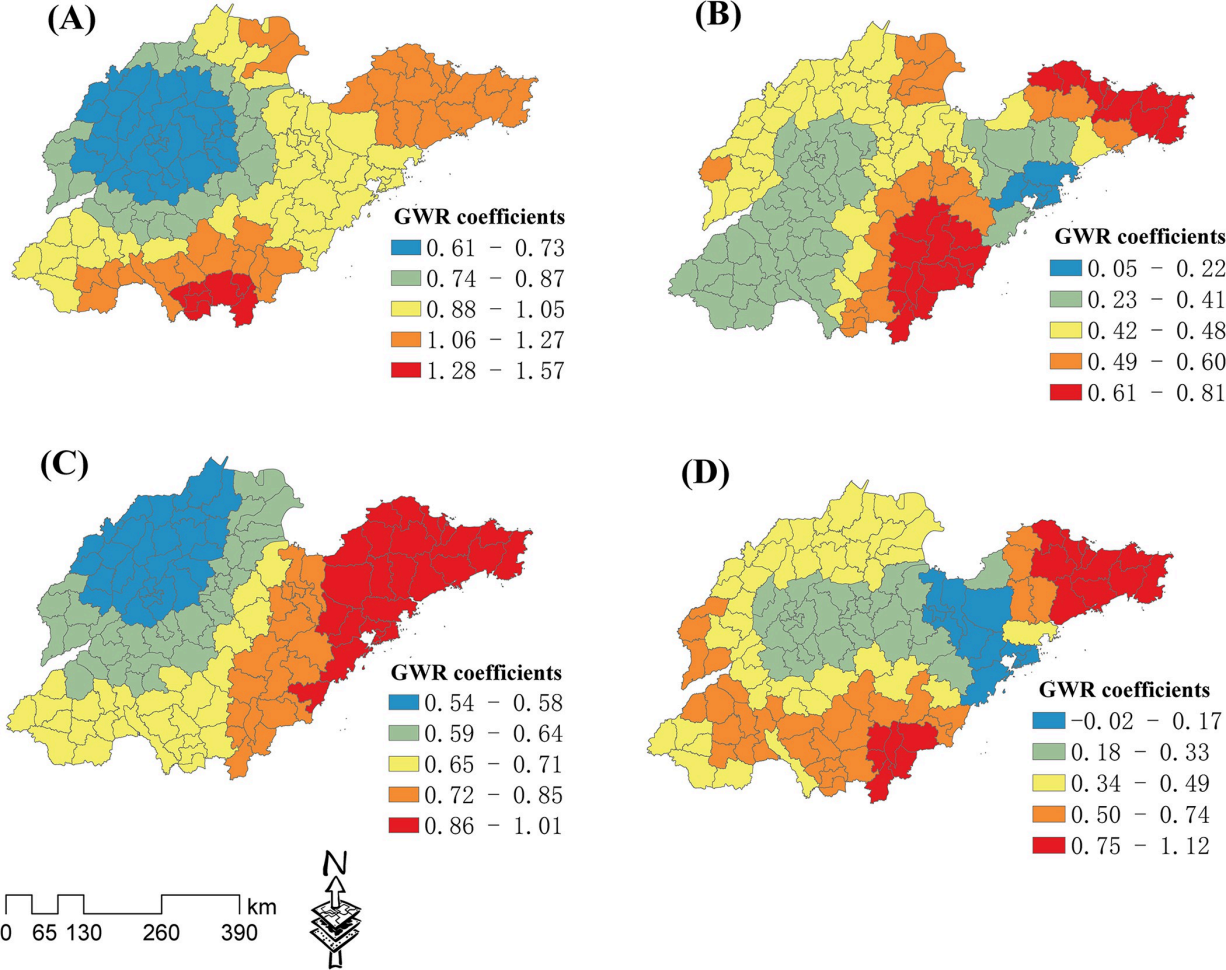
Local GWR coefficients for the processing and manufacturing industry (PM) and key influence factors. Analysis results for (A) gross domestic product (GDP), (B) number of medical facilities (NMF), (C) number of financial institutions (FE), and (D) road density (RD).

The local GWR coefficients of the PM and the key influence factors of GDP, NMF, FE, and RD are shown in **Fig 6**. The regression coefficient value ranges of GDP, NMF, FE, and RD were 0.61–1.57, 0.05–0.81, 0.54–1.01, and -0.02–1.12, respectively, indicating that GDP had the strongest impact on the spatial distribution of the PM. The GDP influence intensity gradually increased from the northwest to the south and east (**Fig 6**A). The NMF had a weak impact on the PM in the western regions and Qingdao, and a strong impact in the southeastern and peninsula regions (**Fig 6**B). The FE positive influence intensity gradually increased from the northwest to the southeast and east (**Fig 6**C). RD positive influence intensity gradually increased from the central regions to the south and east (**Fig 6**D).

**Fig 7:**
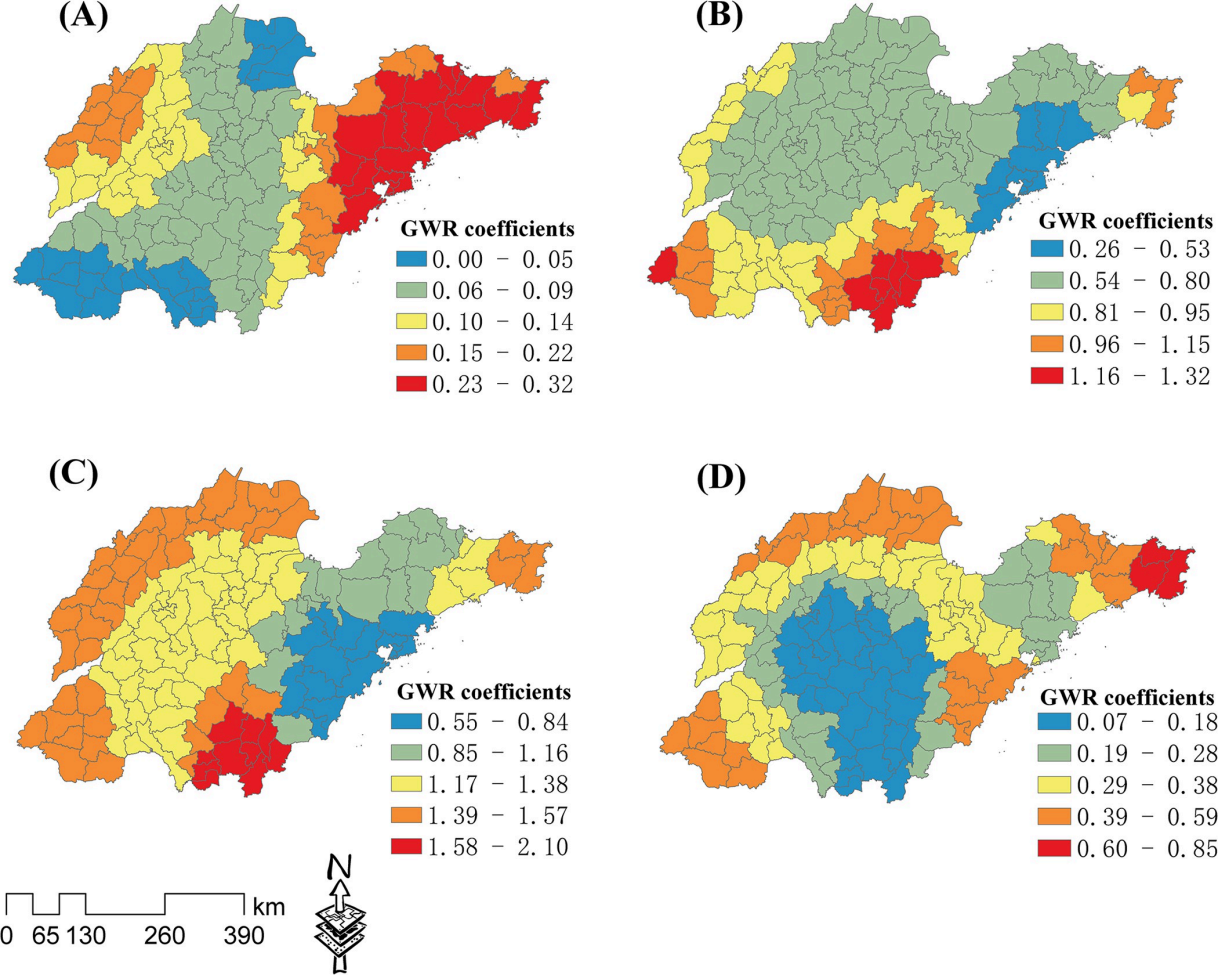
Local GWR coefficients of the high-tech industry (HT) and key influence factors. Analysis results for (A) urbanization rate (UR), (B) number of research institutions (NRI), (C) gross domestic product (GDP), and (D) number of employees in secondary industry (NES).

The local GWR coefficients of the HT and the key influence factors of UR, NRI, GDP, and NES are shown in **Fig 7**. The regression coefficient value ranges of UR, NRI, GDP, and NES were 0–0.32, 0.26–1.32, 0.55–2.1, and 0.07–0.85, respectively, indicating that GDP had the strongest impact on the spatial distribution of the HT. The UR influence intensity gradually increased from the central areas to the eastern and northwestern areas (**Fig 7**A). The NRI influence intensity gradually increased from the most central and northern areas to the south and southwestern areas (**Fig 7**B). The GDP influence intensity gradually increased from the eastern areas of the peninsula to the northwest, southwest, and south (**Fig 7**C). The NES influence intensity gradually increased from the central and southern regions to the surrounding areas **Fig 7**D).

## Discussion

In this study, point-of-interest (POI) data for manufacturing were used to investigate the spatial agglomeration characteristics of different industry types. It is an effective data source for the distribution analysis of spatial elements on a microscale, where corresponding statistical data is difficult to obtain. It is also widely used in industrial spatiotemporal development research [2, 10]. Unlike previous studies, this study aimed to analyze the spatial distribution characteristics of different industry types and their influencing factors. We grouped manufacturing into four industry types: HT, PM, RM, and FLT, and analyzed their spatial agglomeration characteristic and influence factors separately by combining the Getis-Ord Gi* statistical, RF-based importance assessment, and GWR methods.

We found that the four industry types had different agglomeration distribution characteristics, but they all had significant spatial agglomeration characteristics around Qingdao. This is primarily due to Qingdao’s importance as a port city and its ability to attract foreign investments in China [43, 44]. It has obvious advantages in terms of location, policy, and development history. Qingdao held a national leadership position, particularly in the early development of the electronics industry [43]. Qingdao has become the strongest economic city in Shandong after decades of development [45]. Because of the obvious benefits of economic externality, Qingdao has attracted more talent and capital than other regions in Shandong. As a result, the four industry types are generally concentrated in Qingdao.

The RM and FLT also exhibit significant spatial agglomeration characteristics in the vicinity of Linyi city. This is primarily due to the development of China’s transportation network. Linyi is currently located in a strategic transportation hub [46]. It is easily accessible for industrial transfer from the Yangtze River Delta and Beijing Tianjin Hebei regions. Linyi also has the largest population in Shandong. Linyi’s industries, particularly the timber, food, metallurgical, and chemical raw material industries, have grown substantially as a result of obvious advantages of transportation and labor. To some extent, this is consistent with the results of the influence factor analysis; the most important factors influencing the spatial distribution of the RM were NES and RD. Linyi’s proportion of private enterprises is also higher than the national average [39], suggesting that Linyi has a relatively favorable operating environment for private enterprises.

The PM primarily presents strip distribution characteristics along the connecting line between Jinan and Qingdao. This pattern of spatial distribution is similar to that of the Jiaoji railway. The Jiaoji railway was built 100 years ago and has always been an important transportation line within Shandong Province, prompting Shandong’s early development. Shandong has attracted some surplus overseas production capacity from its coastal cities, particularly Qingdao, with the continuous advancement of globalization and opening up since the 1990s. These production capacities spread into inland cities along traffic lines, driving the economic development of cities such as Jinan, Zibo, and Weifang. However, this contradicts the findings of the influence factor analysis. As shown in **Fig 3**C, RD is only the fourth most important factor for the PM; GDP and NMF were the two most important factors influencing PM distribution. This suggests that, in comparison to transportation, economic strength and the level of public service facilities have a greater impact on industry distribution.

The HT exhibits significant spatial agglomeration characteristics in the vicinity of Jinan and Qingdao. The reason for the clustering and distribution in Qingdao has already been discussed. Jinan is the capital of Shandong province, and it houses the majority of the region’s development policymaking institutions. It has the obvious advantage of policy information availability and making communication with relevant institutions easier [47]. The administrative and economic status of the two cities attract significant amounts of capital, research institutions, and talent. As shown in **Fig 3**D, the most important factors for the spatial distribution of the HT are UR, NRI, and GDP. These factors are significantly superior in Jinan and Qingdao compared to those in other regions.

The key spatial influence factors for the four industry types were also different. The NES and RD were most important to the spatial distribution of the RM. The FE and NRI had a greater impact on the spatial distribution of the FLT. GDP and NMF were most important in determining the spatial distribution of the PM. The UR, NRI, and GDP were most important to the spatial distribution of the HT. However, greater factor importance did not imply greater impact intensity. For example, while the NES was the most important RM factor, its GWR coefficient was not the highest. Furthermore, we found that the top four importance factors of all researched manufacturing had a high degree of overlap. They mainly included RD, NES, GDP, FE, NRI, NMF, DRS, and UR. This is somewhat consistent with previous research findings that transportation, labor, economy, business climate, and public service facilities were major factors influencing industry spatial distribution [20]. Natural factors, including WP, VP, and slope, generally had little influence on the distribution of all manufacturing types. In addition, the NDZ also had little impact on the distribution of all manufacturing types. This may be due to the widespread construction of economic development zones in China as part of the economic development policies. In a competitive environment for political achievements, all city governments are striving to develop industrial parks to attract investments [48]. As a result, in terms of the NDZ, it lacks obvious spatial clustering characteristics.

Although this study revealed the spatial distribution features of four industry types and their key influencing factors, it also has some limitations. First, in terms of industry type, this study roughly classified industries into four categories. Additional studies on more detailed industry types are required in future research. Second, this study only analyzed manufacturing spatial distribution for one year. An examination of the manufacturing evolution process over a longer period of time would be more insightful. In future research, we will study the evolution pattern of detailed manufacturing types over a number of years. Third, we only considered the number of enterprises in each manufacturing type and the number of some facilities but did not consider the scale and level of these facilities. The impact of different levels of facilities on the spatial distribution of manufacturing also varies. For example, national-level industrial parks are more appealing to industries compared to local-level industrial parks. Thus, in future research, we will consider the scale and level of relevant facilities.

## Conclusions

This study combined the Getis-Ord Gi* statistic, RF-based importance assessment, and GWR methods to investigate the spatial distribution characteristics of four industry types and their influencing factors. According to the research findings, the RM and FLT were primarily concentrated in the surrounding districts and counties of Linyi and Qingdao. The PM was also primarily concentrated in the surrounding districts and counties of Qingdao, with a few concentrated in the belt regions connecting Jinan, Zibo, and Weifang. The HT was primarily concentrated in the surrounding districts and counties of Jinan and Qingdao.

According to the analysis of the influence factors, the FE and NRI had a significant positive promoting effect on the spatial distribution of the FLT. The GDP and NMF both had significant positive promoting effects on the spatial distribution of the PM. The spatial distribution of HT was significantly influenced by the UR, NRI, and GDP, while the spatial distribution of the RM was significantly influenced by the NES and RD.

## Supporting information

S1 File (ZIP)
